# C1EIP Functions as an Activator of ENO1 to Promote Chicken PGCs Formation via Inhibition of the Notch Signaling Pathway

**DOI:** 10.3389/fgene.2020.00751

**Published:** 2020-07-24

**Authors:** Kai Jin, Dong Li, Jing Jin, Jiuzhou Song, Yani Zhang, Guobing Chang, Guohong Chen, Bichun Li

**Affiliations:** ^1^Key Laboratory of Animal Breeding Reproduction and Molecular Design for Jiangsu Province, College of Animal Science and Technology, Yangzhou University, Yangzhou, China; ^2^Institutes of Agricultural Science and Technology Development, Yangzhou University, Yangzhou, China; ^3^Joint International Research Laboratory of Agriculture and Agri-Product Safety of Ministry of Education of China, Yangzhou University, Yangzhou, China; ^4^Reproductive Medicine Center, The Affiliated Drum Tower Hospital of Nanjing University, Medical School, Nanjing, China; ^5^Animal & Avian Sciences, University of Maryland, College Park, College Park, MD, United States

**Keywords:** chicken PGCs, C1EIP, ENO1, Notch signal, germ cell differentiation

## Abstract

The production of germ cells, especially primordial germ cells (PGCs), is important for avian stem cells and reproduction biology. However, key factors involved in the regulation of PGCs remain unknown. Here, we report a PGC-related marker gene: C1EIP (Chromosome 1 Expression in PGCs), whose activation and expression are regulated by the transcription factor STAT3 (signal transducer and activator of transcription 3), histone acetylation, and promoter methylation. C1EIP regulates PGCs formation by mediating the expression of PGC-associated genes, such as CVH (Chicken Vasa Homologous) and CKIT (Chicken KIT proto-oncogene). C1EIP knockdown during embryonic development reduces PGC generation efficiency both *in vitro* and *in ovo*. Conversely, C1EIP overexpression increases the formation efficiency of PGCs. C1EIP encodes a cytoplasmic protein that interacts with ENO1 (Enolase 1) in the cytoplasm, inhibits the Notch signaling pathway, and positively regulates PGC generation. Collectively, our findings demonstrate C1EIP as a novel gene involved in PGC formation, which regulates genes involved in embryonic stem cell differentiation through interaction with ENO1 and subsequent inhibition of the Notch signaling pathway by the impression of Myc (MYC proto-oncogene).

## Introduction

Primordial germ cells (PGCs), the precursors of sperm and egg, are the only cells with the potential to transmit genetic information to the next generation in developing embryos (Matsui et al., 1992; Han, 2009). In addition, chicken PGCs exhibit unique migration and settling activity compared with mammals, which plays a pivotal role in avian genetic resource protection and animal stem cell research (Burt and Pourquie, 2003; Li et al., 2004; Oishi et al., 2016). Moreover, chicken PGCs function as an alternative and outbred experimental species to humans to compensate for ethical constraints and human germ cell studies (Conti and Giudice, 2008). Notably, multiple genes have been identified to be specifically involved in PGC differentiation, such as *Lin28, Blimp1*, *Prdm14*, and *Stella* (Payer et al., 2003, 2006; Ohinata et al., 2005; Yamaji et al., 2008; West et al., 2009; Childs et al., 2012). Although these genes function as markers to track PGCs, they are not exclusively expressed in PGCs but also other germ cell types (Wei et al., 2008; Macdonald et al., 2010).

Recently, we identified multiple genes and pathways involved in germ cell differentiation and systematically described the role of these pathways and gene function in this process, including BMP4, TGF-BETA, JAK/STAT, Wnt, MAPK, and PPAR (Zhang L. et al., 2015; Zhang et al., 2016; Cheng et al., 2018; He et al., 2018; Zuo et al., 2018b). A new PGC-specific gene, C2EIP (Chromosome 2 Expression in PGCs), which may direct chicken PGC formation via HH (Hedgehog) signaling, has been identified (Zuo et al., 2016, 2018a). Here, we reported another PGC-specific gene, Chromosome 1 Expression in PGCs (C1EIP), which is noticeably expressed in PGCs but not in ESCs (embryonic stem cells) and SSCs (spermatogonial stem cells). We investigated the function of C1EIP *in vitro* and *in ovo* and determined its transcriptional regulating factor (STAT3, DNA methylation, and acetylation). Furthermore, we found that C1EIP can interact with ENO1 to repress the Myc gene via the Notch signaling pathway, which promotes PGC formation in chickens. In conclusion, C1EIP, a critical PGC-specific gene, regulates PGC formation *in vitro* and *in ovo*.

## Materials and Methods

### Materials and Ethics

Chicken eggs were collected from the Rugao Yellow Chickens (Poultry Institute, Chinese Academy of Agricultural Sciences, China) and BALB/c mice were collected from the Experimental Animal Center in Yangzhou University. All eggs were collected and incubated at 37°C at 75% relative humidity for 4.5 and 18 days, respectively. All procedures involving the care and use of animals conformed to the U.S. National Institute of Health guidelines (NIH Pub. No. 85-23, revised 1996) and were approved by the Laboratory Animal Management and Experimental Animal Ethics Committee of Yangzhou University.

### The Identification of C1EIP

The detail of RNA-seq procedure and results was performed as previously reported (Zhang Z. et al., 2015). We analyzed the gene expression pattern in ESCs, PGCs, and SSCs and the gene expression levels were quantified by calculating the RPKM (reads per kilobase transcriptome per million mapped reads) values. The expression of genes with a fold change >2 and FDR (false discovery rate) <0.001 would be filtered as DEGs (differentially expressed genes). The DEGs were divided into six clusters, and cluster 6 is highly expressed in PGCs. Furthermore, the expression analysis showed that the C1EIP was significantly highly expressed in PGCs and little to none in ESCs and PGCs.

### Cell Culture

Procedures for isolating and culturing ESCs, PGCs, and SSCs were performed as previously reported (Zhang Z. et al., 2015). The culturing medium generally contained 43.5 ml of Knockout-DMEM (Gibco, New York, NY, United States, 10829018), 100 μl of gentamicin (Solarbio, Beijing, China, G8170), 0.2 μl of β-mercaptoethanol (Sigma, Missouri, United States, M3148), 200 μl of non-essential amino acids (Sigma, Missouri, United States, M7145), 1 ml of chicken serum (Gibco, New York, NY, United States, 16110-082), 100 μl of SCF (Sigma, Missouri, United States, 300-07-10), 100 μl of bFGF (Sigma, Missouri, United States, F0291), 50 μl of LIF (Millipore, MA, United States, ESG1106), and 500 μl of penicillin (Solarbio, Beijing, China, P1400-100). The detailed constituents in supplemented media for culturing ESCs, PGCs, and SSCs are shown in Supplementary Table S1.

### C1EIP Polyclonal Antibody Production

The pcDNA3.1-C1EIP as the antigen in 1 ml of buffer that contains no chemicals harmful to injection of mouse. Calmly take the mouse out of its cage, reassuringly petting it. Place it on a flat surface for the injection. With a 1-ml syringe, draw in 1 ml of antigen solution. Four subcutaneous injections are done: two on the lower back and two on the thigh. To inject, rub the hair away from the injection site and sterilize with a squirt of ethanol. After the required volume has been injected, let the needle sit for a few seconds, then pull out and gently rub the injection site so nothing leaks out. Repeat for all four sites and place the rabbit back in its cage. Injections will be done every 1 week, with bleeds after the fourth injection. The quality of the antibodies in serum (humoral immune response) of the bleeds is monitored by immunofluorescence (Supplementary Figure S2).

### RA-Induction Model

The PGC induction of the retinoic acid (RA) model was established following a previously described method (Shi et al., 2014). In brief, the chicken ESCs (5 × 10^4^ cell/well) were cultured in 24-well plates in DMEM medium supplemented with 10 mM RA (Solarbio, Beijing, China, IR0060), 15% FBS (Gibco, New York, NY, United States, 26140), and fibronectin (5 μg/cm^2^). Cell morphological changes were visualized using the microscopy every 2 days after the induction.

### Quantitative Real-Time Polymerase Chain Reaction

Tissues and cells were homogenized with TRIzol Reagent, and total RNA was isolated using the RNeasy Mini Kit (Qiagen, Beijing, China, DP424) according to the manufacturer’s instruction. cDNA was produced by a reverse transcription using Revertaid 1st cDNA Synth Kit (Tiangen Biotech, Beijing, China). Quantitative real-time polymerase chain reaction (qRT-PCR) was performed using the FastKing One-Step RT-PCR Kit with SYBR green (Qiagen, Beijing, China, KR123). The mRNA expression of related genes was determined by CFX-Connect Real-time PCR detection system (Bio-Rad, CA, United States, 7500Fast). Data were quantified relative to the housekeeping gene β*-Actin* using the 2^–ΔΔCt^ method. Sequences of primers used in qRT-PCR are listed in Supplementary Table S2.

### Construction of C1EIP Promoter Deletion and Mutant Fragments

Based on bioinformatics analysis of the C1EIP promoter in Rugao yellow chickens, we fixed the downstream primer, changed the upstream primer, and designed primers to amplify different deletion fragments. The mutant’s primers were designed as the Manual of Fast Mutagenesis System Kit (Abbexa, Cambridge, United Kingdom, abx098073) and amplify different mutant fragments. The primers of each deletion and mutant fragments are shown in Supplementary Tables S3, S4.

We constructed pGL3 and pEGFP-N1 vector, cleaved the vectors and PCR products with dual enzymes, recovered the cleaved products, and ligated them. After confirming enzymes by enzymatic cleavage and sequencing, these deletion and mutant fragments were named and used in the next experiments.

### Dual−Luciferase Activity Detection

DF-1 cells were transfected with plasmids of wild-type or mutant C1EIP promoter using Lipofectamine TM 3000 transfection reagent. Then, those well-grown DF-1 cells were cultured in 24-well plates at a starting density of 2 × 10^5^ cells. The dual luciferase expression vectors or PGL3-Basic vector as a control was mixed with pRLSV40 at a ratio of 1:30 and used to label DF-1 cells with luciferase. After 24–48 h of co-transfection, cells were harvested, and the promoter activity was tested by using the dual luciferase reporter gene detection system according to the manufacturer’s instruction. The data were represented as firefly/renilla luciferase activity ratio.

### Construction of Interference and Overexpression Vectors

According to the coding region sequence of chicken C1EIP gene, three shRNAs targeting chicken C1EIP were designed and separately inserted into the packaging vector (pGMLV-SC5), which contains the gene encoding green fluorescent protein (GFP). The open reading frame (ORF) of C1EIP was amplified by PCR. Purified PCR products were ligated with the pcDNA3.0(+) vector and C1EIP overexpression plasmid was confirmed by sequencing. The detailed information of primers used in this study are listed in Supplementary Table S5.

### Flow Cytometry Analysis

Cells harvested from different induction days were blocked using blocking buffer (PBS containing 10% fetal bovine serum) (Gibco, New York, NY, United States, 10270-106) for 2 h at 37°C. Single-cell suspension was made in L-15 medium (Sigma catalog L-99002) with a cell concentration of 1 × 10^7^ cells/ml. Cell samples were mixed gently with appropriately diluted labeled antibodies (CVH, Abcam, Cambridge, United Kingdom, ab13840, 1:100; CKIT, Thermo Fisher Scientific, Shanghai, China, 14-1172-81, 1:100) and incubated at 4°C for 30 min. Then, cells were washed three times with PBS containing 0.1% Tween-20 (Solarbio, Beijing, China, T8220), followed by the incubation with fluorescence coupled secondary antibodies [Goat Anti-Rabbit IgG FITC Conjugated, CWBIO, Shanghai, China, CW0114S, 1:100; Goat Anti-Mouse IgG H & L (TRICT), Abcam, Cambridge, United Kingdom, ab6786, 1: 100] at 37°C in the dark for 2 h. Then, cells were washed three times with PBS containing 0.1% Tween-20. The staining signal was analyzed by FACS LSRFortessa (BD Biosciences, United States) with a minimum of 10,000 events in each experiment.

### Immunofluorescent Staining

Cells were fixed with 4% paraformaldehyde for 30 min at room temperature and permeabilized with 0.5% Triton X-100 (Solarbio, Beijing, China vT8200) for 10 min. Samples were then blocked with blocking buffer (PBS containing 10% fetal bovine serum) at 37°C for 2 h or 4°C overnight. Samples were incubated with diluted primary antibodies in blocking buffer at 37°C for 2 h or 4°C overnight. After the primary antibody incubation, samples were washed three times with PBS containing 0.1% Tween 20 (Solarbio, Beijing, China, T8220) followed by the incubation with fluorescence-conjugated secondary antibodies diluted in blocking buffer for 2 h at 37°C. Then samples were washed three times with PBS containing 0.1% Tween 20. Nuclei were counterstained with DAPI (Beyotime, Beijing, China, C1002). Images were obtained using a confocal microscope (Olympus, Tokyo, Japan, FV1200). Primary antibodies included anti-CVH (Abcam, Cambridge, United Kingdom, ab13840, 1:100), anti-CKIT (Invitrogen, CA, United States, 14-1172-81, 1:100), anti-C1EIP (polyclonal antibody, 1:10), and anti-HA (Abcam, Cambridge, United Kingdom, ab187915, 1:100). Secondary antibodies included goat anti-Rat IgG (Proteintech, Chicago, United States, SA00003-11, 1:1000, [FITC] labeled) and goat anti-mouse IgG (Proteintech, Chicago, United States, SA00003-12, 1:1000, [TRITC] labeled).

### Periodic Acid Schiff (PAS) Glycogen Staining

Sections of genital ridge were deparaffinized and rehydrated, followed by a 0.5% periodic acid solution rinse for 10 min. Sections were then placed in Schiff reagent for 15 min (sections turned into a light pink color during this step). Then, these sections were rinsed with running water for 5 min (immediately sections turned into a dark pink color) and counterstained in hematoxylin for 30 s. After a tap water wash for 5 min, sections were dehydrated and mounted with coverslips.

### GST Pull-Down and Co-IP

The GST pull-down and Co-IP experiment method is described in the previous paper published by our lab (Zuo et al., 2018a). In brief, the full-length CDS of C1EIP was ligated into the Pet-49(b)-GST plasmid, and the experimental plasmids were transformed into the *Escherichia coli* BL expression strain. Expression was induced by IPTG at 18°C, and the protein was isolated from cells, purified from the precipitate, concentrated, and used in GST pull-down experiments to identify interactions. To prepare samples for MS analysis, protein was alkylated and enzymatically cleaved after GST pull-down, and the sequence of interacting proteins was identified by LC-MS/MS.

The DF-1 cell was transfected by different groups: pcDNA3.0-ENO1-HA+pEGFP-C1EIP, pcDNA3.0-ENO1-HA, and pEGFP-C1EIP. After 48 h, the cells were lysed in RIPA buffer and centrifuged at 13,000 × *g* at 4°C for 15 min. The protein was stored at −70°C. For Co-IP, the extracted protein was incubated overnight with an optimal concentration of anti-HA antibody. The protein was then centrifuged at 1000 × *g* at 4°C for 10 s and for Western blot analysis.

### Data Analysis

The SRA accession numbers for the RNA-seq data reported in this paper are as follows: SRR3720923, SRR3720924, and SRR3720925. The expression analysis by Bowtie2 and SamTools was based on the reference genome Galgal5.0 (UCSC). The expression pattern was analyzed by R (3.0.1.) and visualized by IGV (2.6.3.). The protein interaction was analyzed via PCViz website^1^ and GeneMANIA website^2^. Statistical analyses were performed with Student *t* test using SPSS22.0 software package. *p* < 0.05 and <0.01 were considered significant and highly significant, respectively. Charts and diagrams were prepared using GraphPad Prism 6 and R studio.

## Results

### C1EIP Is Highly Expressed in PGCs and Located in the Cytoplasm

**FIGURE 1:**
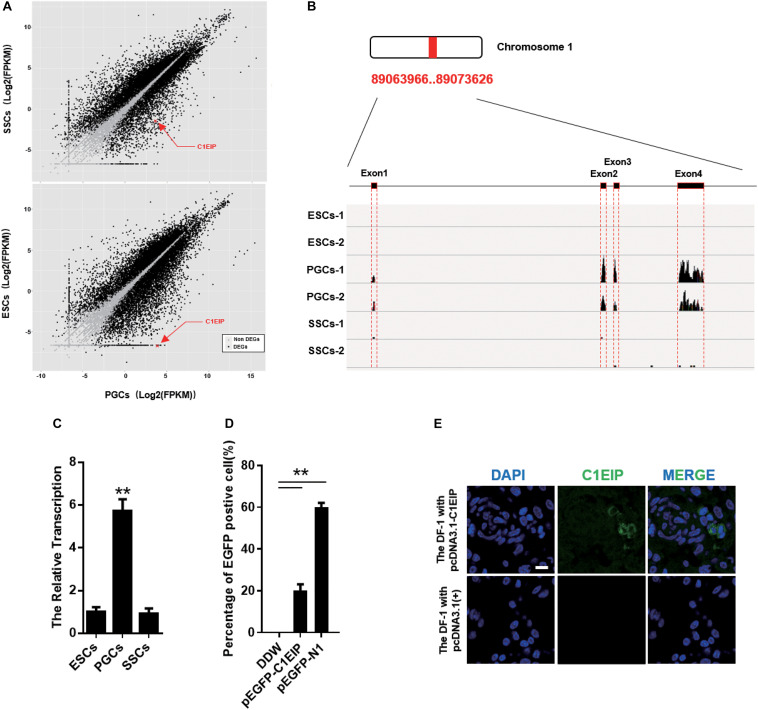
C1EIP highly expressed in PGCs and located in cytoplasm. **(A)** The distribution of differentially expressed genes in ESCs, PGCs, and SSCs indicates that C1EIP is highly expressed in PGCs. Red represents upregulated genes, green represents downregulated genes. **(B)** The sequencing coverage of C1EIP in ESCs, PGCs, and SSCs. **(C)** qRT-PCR analysis of *C1EIP* expression in ESCs, PGCs, and SSCs. **(D)** Confirmation of C1EIP-EGFP fusion protein expression. Cells were transfected with pEGFP-N1 vector as a positive control, or mock-transfected with double-distilled water (DDW) as a negative control. **(E)** Indirect immunofluorescence showing cytoplasmic localization of C1EIP. Cells transfected with pcDNA3.1(+) vector were used as a negative control. Scale bar: 10 μm (data are shown as mean ± SEM and Student *t*-test were utilized for statistical analysis; *p < 0.05, **p < 0.01).

Primordial germ cells are ancestral cells of sperm and eggs, and our lab has previously described their migration, colonization, and differentiation (Xiao et al., 2004; Li et al., 2008). Based on previous data from our lab (Zhang Z. et al., 2015), we identified the PGC-specific gene: C1EIP (XM_416629.4, Chr.1) (Figure 1A) (Supplementary Figures S1A–C). The RNA-seq results show significantly higher expression of C1EIP in PGCs compared with ESCs and SSCs (Figure 1B). Consistently, the mRNA level of C1EIP was fivefold higher in PGCs than that in ESCs and SSCs (Figure 1C). In order to characterize the capacity of C1EIP protein coding, a pEGFP-N1-C1EIP vector was constructed with a mutated stop codon of C1EIP and transfected into DF-1 cells. pEGFP-N1-C1EIP successfully encoded protein in DF-1 cells with expression of EGFP-tagged C1EIP protein (Figure 1D and Supplementary Figures S2A,B). To further investigate the subcellular localization of C1EIP, a polyclonal antibody was prepared using a constructed pcDNA3.0-C1EIP vector and injected the vector into mice intraperitoneally. The polyclonal antibody titer was 1:10, and this antibody is available to identify C1EIP specifically (Supplementary Figures S2C–E). The pcDNA3.0-C1EIP vector was also transfected into DF-1 cells. C1EIP was expressed in the cytoplasm of DF-1 cells according to immunofluorescence assay (Figure 1E). Notably, we characterized a PGC-specific gene that encodes a cytoplasmic localized protein.

### C1EIP Is Highly Expressed in PGCs via STAT3 and Histone Modification

**FIGURE 2:**
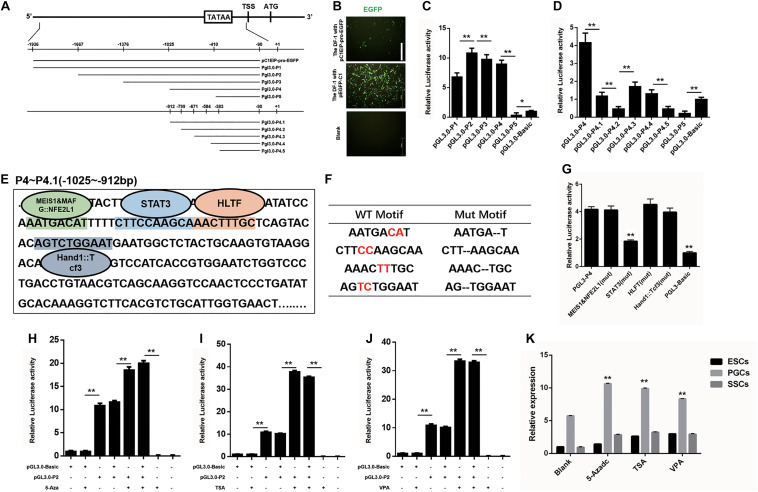
C1EIP is mediated by Transcription factor STAT3 and histone modification. **(A)** Schematic diagram of C1EIP promoter fragment cloning and vector construction. **(B)** EGFP expression in DF-1 cells after transfection with pC1EIP-pro-EGFP, indicating that the cloned C1EIP promoter is active. DF-1 cells transfected with empty and pEGFP-N1 vector were used as negative and positive controls, respectively. Scale bar: 100 μm. **(C)** Luciferase activity in DF-1 co-transfected with pGL3-P1, pGL3-P2, pGL3-P3, pGL3-P4, and pGL3-P5. DF-1 cells transfected with pGL3-basic was a negative control. **(D)** Luciferase activity in DF-1 co-transfected with pGL3-P4, pGL3-P4.1, pGL3-P4.2, pGL3-P4.3, pGL3-P4.4, pGL3-P4.5, and pGL3-P5. DF-1 cells transfected with pGL3-basic was a negative control. **(E)** The location of transcription factor in the core region of the C1EIP promoter. The corresponding binding site (parallelism color) of transcription factor (MEIS1&MAFG:NFE2L1, STAT3, HLTF and Hand1::Tcf3) in the core region (–1025 to –912 bp) of the C1EIP promoter. **(F)** Table of transcription factor binding site and mutation. **(G)** The double luciferase reporter system was used to assess C1EIP promoter activity when the transcription factor binding site was mutated (Mut). **(H)** The double luciferase reporter system was used to examine the effects of 5-Aza on C1EIP promoter activity. **(I)** The double luciferase reporter system was used to examine the effects of TSA on C1EIP promoter activity. **(J)** The double luciferase reporter system was used to examine the effects of VPA on C1EIP promoter activity. **(K)** Effect of DNA methylation and histone acetylation on expression of C1EIP in ESCs, PGCs, and SSCs (data are shown as mean ± SEM and Student *t*-test was utilized for statistical analysis; *p < 0.05, **p < 0.01).

The spatiotemporal variation of genes is largely regulated by the activity of gene promoters (Busby and Ebright, 1994; Fickett and Hatzigeorgiou, 1997). To investigate the transcription factor of the C1EIP, we identified the TATA box and CAAT box to determine the promoter region of C1EIP. The sequence (−1936 to −90 bp) of C1EIP promoter was cloned and inserted into the pEGFP-C1EIP recombinant vector (Figure 2A). The expression of EGFP was detected in DF-1 cells transfected with this pEGFP-C1EIP recombinant vector. The results showed that the C1EIP promoter facilitates EGFP expression in DF-1 cells, confirming the activity of C1EIP promoter (Figure 2B).

The region (−1025 to −912 bp) was identified as the core region of the C1EIP promoter based on the luciferase activity determined by two truncated fragment group experiments (Figures 2A,C,D). Moreover, binding sites for transcription factors involved in stem cell maintenance and differentiation such as MEIS1&MAFG:NFE2L1, STAT3, HLTF, and Hand1::Tcf3 are located at this core region of the C1EIP promoter (Figure 2E). We detected the activity of the core C1EIP promoter with mutations at each transcription factor binding site and results revealed STAT3 to be a positive regulating factor, whereas others did not show significant effect (Figures 2F,G).

The epigenetic factors (DNA methylation and histone acetylation) also affect the activity of promoters (Eden et al., 1998; Vaissière et al., 2008; Rivière et al., 2011). Pyrophosphate sequencing results identified no CpG island in the C1EIP promoter region but the methylation inhibitor 5-Aza (5-Azacitidine, a DNA methyltransferase inhibitor) significantly enhances (*p* < 0.01) activity of the pGL3.0-P2 vector. Although C1EIP does not contain a CpG island, its expression was regulated by the methylation of a region other than the core area (Figure 2H). Furthermore, the addition of TSA (trichostatin A, a histone deacetylase inhibitor) and VPA (valproic acid, a histone deacetylase inhibitor) significantly increased the activity of the C1EIP promoter, suggesting that C1EIP expression was positively regulated by histone acetylation (Figures 2I,J). Consistent qRT-PCR results were also observed in ESCs, PGCs, and SSCs (Figure 2K).

Taken together, our results indicate that STAT3 binding, histone acetylation, and promoter methylation regulate C1EIP expression based on C1EIP promoter activity.

### C1EIP Promotes PGC Formation *in vitro* Through Limiting Expression of Pluripotency Gene and Activating PGC Differentiation Genes

**FIGURE 3:**
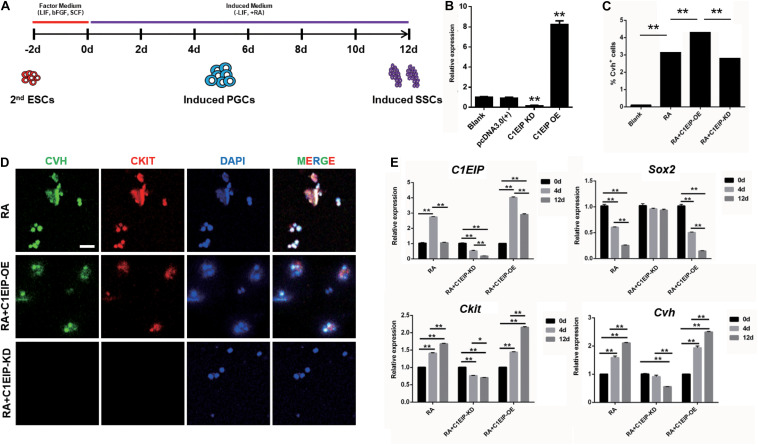
C1EIP enhances the PGC generation *in vitro*. **(A)** Schematic diagram of RA induction model in chicken male germ cell deafferentation. **(B)** Expression of C1EIP mRNA in the different RA induction models was detected by qRT-PCR. C1EIP OE and KD groups represent ESC transfected with C1EIP overexpression and knockdown vectors, respectively. ESC mock-transfected with no plasmid was the blank control. **(C)** The percentage of CVH-positive cells by flow cytometry. **(D)** Fluorescence microscopy after 4–6 days showing PGC-like CVH/CKIT-positive cells. ESC with RA induction and no transfection was the control, scale bar: 50 μm. **(E)** qRT-PCR was used to quantify *C1EIP*, *Cvh*, *Ckit*, and *Sox2* expression after C1EIP knockout or overexpression (data are shown as mean ± SEM and Student *t*-test was utilized for statistical analysis; *p < 0.05, **p < 0.01).

To study the function of C1EIP in PGC generation, we implemented a model for inducing ESC differentiation into SSCs by *in vitro* RA treatment described previously by our lab (Figure 3A) (Shi et al., 2014). C1EIP was knocked down (shRNA) and overexpressed using lentiviruses vector (Figure 3B and Supplementary Figure S3). CVH (chicken vasa homologous) is a key factor involved in PGC development. Flow cytometry analysis shows increased number of CVH^+^ cells in the C1EIP-overexpressing group and a decreased number of CVH^+^ cells in the C1EIP-knockdown group (Figure 3C and Supplementary Figure S4A). Consistently, overexpression of C1EIP promotes the formation of PGC-like cells; conversely, the PGC-like cells were decreased with knockdown (Figure 3D and Supplementary Figure S4B).

To further dissect the mechanism of the role of C1EIP in PGC differentiation *in vitro*, we determined the expression of marker genes, the downregulation of the pluripotency-related gene *Sox2* and upregulation of the PGC marker genes (*Ckit* and *Cvh*) were observed under overexpression of C1EIP, along with no change in SSC marker gene expression (*Integrin a6*, *Integrin b1*, and *Stra8*) and the germ cell marker gene *Dazl* compared with the RA treatment group. In contrast, C1EIP knockdown significantly decreased mRNA levels of *Ckit* and *Cvh* while it increased that of *Sox2* compared with RA treatment group (Figure 3E and Supplementary Figure S4C).

Conclusively, these data demonstrated that C1EIP promotes PGC formation *in vitro* via the inhibition of pluripotency gene expression and the activation of PGC differentiation gene expression.

### C1EIP Promotes PGC Formation *in ovo*

**FIGURE 4:**
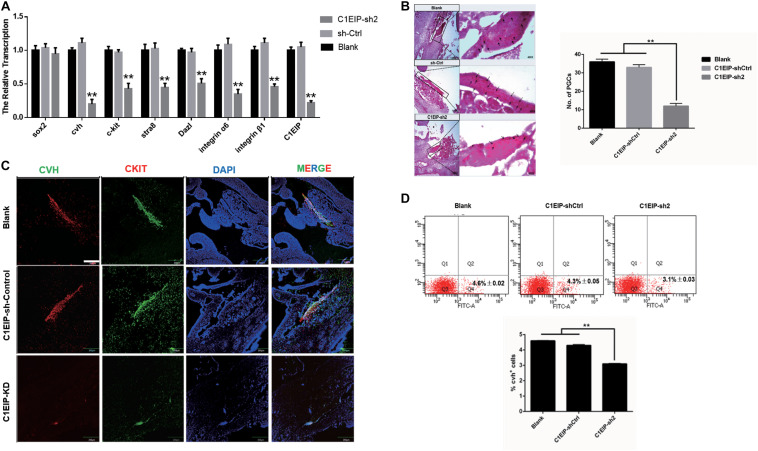
C1EIP enhances the PGC generation *in ovo*. **(A)** Gene expression of marker gene was quantified by qRT-PCR. **(B)** PAS staining of day 4.5 chicken embryo paraffin section in each group. Black arrows represent PGCs. **(C)** Immunofluorescence of the genital ridge in control and C1EIP knockdown (KD) hatching chicken embryos. Chicken embryos without any treatment were used as controls. Scale bar: 240 μm. **(D)** CVH-positive cells were quantified using flow cytometry in the indicated groups. Chicken embryos without any treatment were used as blank controls (data are shown as mean ± SEM and Student *t*-test was utilized for statistical analysis; *p < 0.05, **p < 0.01).

To determine the function of C1EIP *in ovo*, we further mixed the knockdown vectors isovolumetrically with polyethylenimine and then injected them into chick embryos at day 2.5. EGFP expression was visualized in embryonic and heart sections, suggesting that C1EIP knockdown negatively affected chick embryo development and the vector could be expressed in embryo (Supplementary Figures S5A,B). qRT-PCR, immunohistochemical assay and PAS results show reduced expression of CVH and CKIT in the knockout group compared to the control group in genital ridges (Figures 4A–C). Moreover, flow cytometry analysis results show less numbers of PGCs in the knockdown groups (Figure 4D). All these data demonstrated that C1EIP played a critical role in PGC formation *in ovo*.

### C1EIP Functions as an Activator of ENO1 via Notch Signaling to Promote Chicken PGC Formation

**FIGURE 5:**
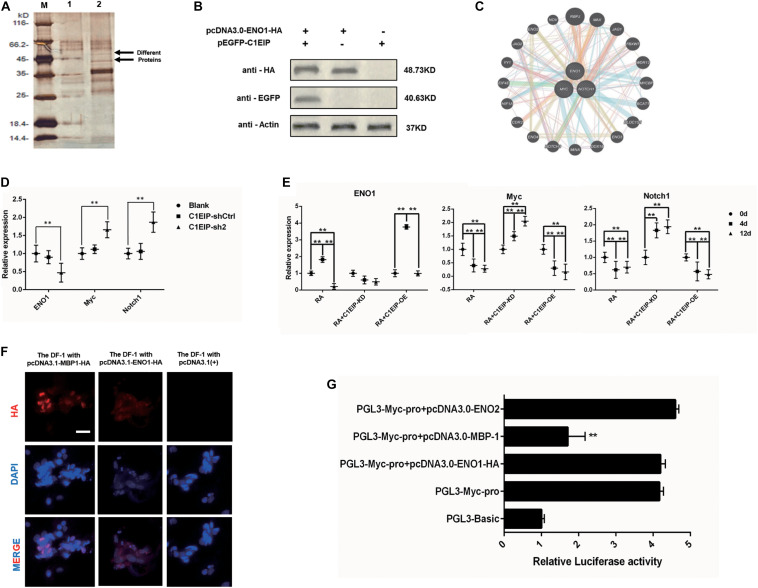
C1EIP as activation of ENO1 via Notch signal to promote the chicken PGC formation. **(A)** Silver staining of GST pull-down samples. There were two distinct protein bands in the experimental groups. M: Marker; 1: Control group; 2: Experimental group. **(B)** Co-immunoprecipitation assay for the interaction between ENO1 and C1EIP; the expression of the C1EIP-EGFP fusion protein was not detected after immunoprecipitation with HA antibody in pcDNA3.0-ENO1-HA or pEGFP-C1EIP. The expression of the C1EIP-EGFP fusion protein was detected in the ENO1-HA interaction protein complex precipitated by HA antibody in pcDNA3.0-ENO1-HA and pEGFP-C1EIP co-transfection. **(C)** ENO1 co-expression network diagram; the interaction between the ENO1 and the Myc and Notch1 was found by online analysis software PCViz and GeneMania. **(D)** Related genes mRNA expression changes *in ovo*; there was no significant difference in the expression levels of ENO1, Myc, and Notch1 in the blank group and control group; however, the expression of ENO1 was significantly decreased after knockdown of C1EIP, while the expression of Myc and Notch1 was significantly upregulated. **(E)** Related genes mRNA expression changes *in vitro*; ENO1 expression on d4 was higher than that on d0 and d12. Overexpression of C1EIP resulted in a further increase in ENO1 expression on d4, but significantly reduced the expression of ENO1 after C1EIP knockdown. The expression of Myc and Notch1 was decreased in normal RA induction. The expression of Myc and Notch1 was downregulated after overexpression of C1EIP, but they were significantly increased after knockout of C1EIP. **(F)** Indirect immunofluorescence showing cytoplasmic localization of ENO1 and MBP-1. Cells transfected with pcDNA3.1(+) vector were used as a negative control. Scale bar: 30 μm. **(G)** Activity analysis of Myc promoter, compared with the PGL3-Basic transfection control group; the dual luciferase activity of PGL3-Myc-pro vector was significantly upregulated. However, PGL3-Myc-pro did not significantly change the activity of Myc promoter after co-transfection with pcDNA3.0-ENO1-HA and pcDNA3.0-ENO2, respectively, and the activity of Myc promoter after co-transfection with pcDNA3.0-MBP-1 decreased significantly, *n* = 3 (data are shown as mean ± SEM and Student *t*-test was utilized for statistical analysis; *p < 0.05, **p < 0.01).

We investigated the molecular mechanism underlying C1EIP-mediated PGC generation by characterizing C1EIP-ENO1 interactions via GST pull-down assay (Figure 5A). Moreover, Co-IP experiments also demonstrated the interaction between C1EIP and ENO1 (Figure 5B). Further analysis of the protein interaction network with PCViz and GeneMANIA database revealed the direct regulatory relationship between ENO1 and Myc as well as Myc and Notch1 (Figure 5C). Myc and Notch1 are important signaling molecules in the Notch signaling pathway, which is a crucial pathway associated with the formation of PGCs (Zuo et al., 2018b). C1EIP and ENO1 might affect PGC formation through the Notch signaling pathway. Knockdown of C1EIP *in vitro* and *in ovo* significantly decreases *ENO1* expression, while the expression of Myc and Notch1 is significantly increased (Figures 5D,E). This shows that expression of C1EIP and ENO1 changes simultaneously, while Myc and Notch1 are opposite, indicating that C1EIP and ENO1 play a positive role in regulating male germ cell differentiation, while Myc and Notch1 play a negative role effect (Figures 5D,E).

However, while Myc is mainly located in the nucleus, C1EIP and ENO1 are localized in the cytoplasm. We discovered that the *ENO1* gene encodes two different proteins, which are the full length ENO1 protein and the MBP-1 protein missing 96 amino acid residues. Immunofluorescence showed that MBP-1 was expressed in nucleus (Maranto et al., 2015). Moreover, the MBP-1 protein binding site is found in the MYC promoter region (Feo et al., 2000), and experiments show that MBP-1 can inhibit the activity of the MYC promoter (Figures 5F,G). Thus, C1EIP might regulate PGC formation by the inhibition of Notch signaling pathway through ENO1, which could be transferred into MBP1 and then downregulate Myc.

## Discussion

**FIGURE 6:**
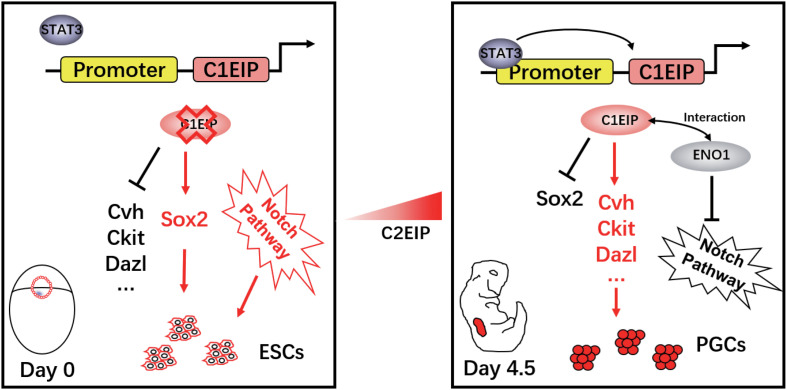
Schematic diagram of C1EIP as an activator of ENO1 to promote chicken PGC formation via inhibition of the Notch signaling pathway.

Here, we describe that C1EIP is a new marker gene of PGCs, which is specifically expressed in the cytoplasm of PGCs. Knockdown of C1EIP blocks the formation of PGCs both *in vitro* and *in ovo*, while overexpression of C1EIP promotes the generation of PGCs. Moreover, C1EIP expression is regulated by epigenetic factors (methylation and acetylation) and its transcription factor STAT3. Here, we report that C1EIP acts as a chicken PGC marker gene, and its expression improves the efficacy of PGC formation *in vitro* and *in ovo* (Figure 6).

The process of PGC formation is regulated by multiple genes. For example, the generation and proliferation of PGCs are regulated by factors such as BMP4 and NANOS2 (Lawson et al., 1999; Saga, 2010; Shirazi et al., 2012; Oulhen et al., 2019). Specifically, CVH and CXCR4 can be used to trace the migration process of PGCs (Noce et al., 2001; Lee et al., 2017). Moreover, some genes like Prdm14 and Blimp1 are also considered to be crucial factors in PGC formation and differentiation (Ohinata et al., 2005). However, these marker genes are also widely expressed in other germ cells, like ESCs and SSCs, which limits the marker potential of these genes. As the low efficiency of PGC formation *in vitro* and *in vivo* grandly restricts the application of PGCs, we urgently need to explore the specific factor involved in PGC formation. In the previous study, we screened two candidate genes (C1EIP and C2EIP) via high-throughput sequencing. These two genes are specifically expressed in PGCs and exhibit the potential to be PGC marker genes, which provides a new idea to improve the formation efficiency and application of PGCs. C2EIP has been proven as a new marker for PGCs, which significantly activates the formation of PGCs *in vitro* and *in ovo* by interacting with PTCH2 to activate the HH signaling pathway (Zuo et al., 2018a). To further understand the mechanism of PGC formation, we continue to investigate the potential of C1EIP as a new marker gene for PGCs, not only because of their similar expression patterns but more importantly due to the distinctive mechanism under the role of C1EIP. In this work, we are trying to identify a novel marker for PGCs and potentially find an efficient approach to promote the PGC generation.

C1EIP, especially in PGCs and promoter, is the most important factor involved in the regulation of the gene expression. Currently, the epigenetic modification (such like DNA methylation, histone acetylation, and transcription factors) is regarded as the main factor affecting the activity of gene promoter (Danino et al., 2015). DNA methylation is an epigenetic modification and mainly occurs in the CpG nucleotide-rich region, which can interfere with transcription factor binding, which regulates gene transcription (Jones and Liang, 2009; Deaton and Bird, 2011). Although genome-wide studies have revealed patterns of DNA methylation throughout the genome of CpG island and CpG island-free promoters, the role of DNA methylation in CpG island-free promoters is often overlooked (Rakyan et al., 2008). At present, many genes without CpG islands in the promoter region have been reported to exhibit DNA methylation in normal tissues (Eckhardt et al., 2006). Han et al. found that treatment of 5-Aza on LAMB3 and RUNX3P1 genes without CpG islands in the promoter region can directly lead to the transcriptional silencing and subsequent gene silencing *in vitro* (Han et al., 2011). In our research, CpG islands do not exist in the C1EIP promoter region. After the treatment of 5-Aza, the C1EIP promoter activity and expression were enhanced in PGCs with the involvement of DNA methylation in the transcriptional regulation of C1EIP. This preliminarily proved that DNA methylation occurs in the promoter region even without CpG islands. There was no significant change of C1EIP expression after 5-Aza treatment in other germ cells without or with low C1EIP expression, such as ESCs and SSCs, indicating that C1EIP expression in ESCs and SSCs may require other transcriptional regulatory elements, which are present in PGCs but not present in ESCs and SSCs. Except for DNA methylation, acetylation also can regulate gene transcription by mediating the repulsive power of histones and the interaction of proteins (Li et al., 2014). De Silva et al. reported that the treatment of TSA to induce CD4^+^ T cells increased SAMHD1 expression (de Silva et al., 2013). Teng et al. (2010) found enhanced activity of mouse Oct4 promoter after adding VPA. In this study, C1EIP expression in ESCs, PGCs, and SSCs was significantly enhanced with TSA or VPA treatment. Consistently, these findings indicate that acetylation is involved in the transcriptional regulation of C1EIP.

STAT3 is an important member of the signal transducers and activators of transcription (STAT) family, which can negatively regulate helper T cell (Th1)-mediated inflammatory response as well as activate a variety of immunosuppressive genes (Welte et al., 2003; Kortylewski et al., 2005). In the reproduction process, STAT3, as an important downstream signaling molecule in the JAK-STAT signaling pathway, induces phosphorylation (Wawersik et al., 2005). Then the phosphorylated STAT3 translocates to the nucleus and mediates the signal transduction of the JAK-STAT signaling pathway, thereby indirectly regulating the male germ cell differentiation (Issigonis et al., 2009; Sheng et al., 2009). In this study, the STAT3 binding site was identified between −1025 and −912 bp in the core region of the C1EIP promoter and characterized to regulate C1EIP gene transcription. It is possible that the nuclear translocation of STAT3 followed by its phosphorylation initiates the transactivation ability of STAT3. Notably, the mechanism of PGC origin remains elusive. As a vital upstream factor of C1EIP, STAT3 may play a critical role in the early embryonic development. Thus, exploring the role of STAT3 in regulating C1EIP gene expression helps us to further understand the mechanism of chicken PGC origin.

Notch signaling pathway has extensive and multiple effects on cell growth and development, mainly involved in cell proliferation, differentiation, apoptosis, and adhesion, especially in germ cell differentiation (Leong and Karsan, 2006). Dallas et al. have shown that Notch signal negatively regulated the microecology of male germ stem cells in Drosophila (Vanorny and Mayo, 2017). Braydich-Stolle found that GDNF upregulated NUMB expression during the maintenance of SSCs, and degradation and suppression of Notch target genes suggested that Notch signaling may affect SSC formation in rats (Braydich-Stolle et al., 2005). These evidences indicate that the Notch signaling pathway plays a negative regulatory role in the process of germ cell development. Previous studies have reported Myc as a downstream target gene of Notch1 (Korkaya and Wicha, 2009), which was also found in the ENO1 gene protein network interaction analysis in our study. Inhibiting Myc can effectively block the function of the Notch signaling pathway (Klinakis et al., 2006; Palomero et al., 2006; Weng et al., 2006). As a reprogramming factor, Myc is highly expressed in early stage germ cells like ESCs or cancer cells while it exhibits low expression in germ cells, such as PGCs and SSCs, and somatic cells. In this work, we systematically speculated and clarified the mechanism under the role of C1EIP in PGC formation: the interaction of highly expressed C1EIP and ENO1 promotes the translocation of ENO1 into the nucleus, in which ENO1 binds to the promoter region of Myc and then negatively regulates the transcription and expression of Myc, thereby inhibiting the NOTCH signaling pathway.

In summary, we present a novel chicken PGC-specific marker gene, C1EIP, and demonstrate its critical role in the generation of PGCs, presenting a new potential to improve the efficiency of PGC formation *in vitro* and *in ovo*. The early expression of C1EIP is co-regulated by DNA methylation, histone acetylation, and STAT3, which provides a theoretical foundation for further investigating the origin of PGCs. During the process of PGC formation, C1EIP enhances ENO1/BMP1 activity and then inhibits Myc expression to facilitate PGC formation via Notch signaling. This revealed the role of Notch signaling pathway in the development of chicken PGCs. These findings together present solid evidences that the C1EIP exhibits a great potential working as a marker gene in the future to help improve the generation and application of chicken PGCs.

## Data Availability Statement

Publicly available datasets were analyzed in this study. This data can be found here: The SRA accession numbers for the RNA-seq data reported in this paper are: SRR3720923, SRR3720924, and SRR3720925.

## Ethics Statement

The animal study was reviewed and approved by animal experiments were approved by the Institutional Animal Care and Use Committee of the Yangzhou University Animal Experiments Ethics Committee (permit number: SYXK [Su] IACUC 2012-0029). Written informed consent was obtained from the owners for the participation of their animals in this study. All experimental procedures were performed in accordance with the Regulations for the Administration of Affairs Concerning Experimental Animals approved by the State Council of the People’s Republic of China.

## Author Contributions

KJ and BL: conceptualization. JJ: methodology. JS: software. KJ, DL, and JJ: validation. DL: formal analysis. GBH: investigation. DL: resources. KJ: data curation, writing – original draft preparation, and visualization. YZ: writing – review and editing. GHC and BL: supervision, project administration, and funding acquisition. All authors contributed to the article and approved the submitted version.

## Conflict of Interest

The authors declare that the research was conducted in the absence of any commercial or financial relationships that could be construed as a potential conflict of interest.

## Footnotes

1 https://www.pathwaycommons.org/

2 https://genemania.org/

## Supplementary Material

FIGURE S1 The C1EIP highly expressed in PGCs. **(A)** Transcriptome analysis of ESCs, PGCs, and SSCs. The differential expression of genes in these cells divided into six clusters. **(B)** The PCA analysis of C1EIP in ESC, PGC, and SSC. **(C)** The line chart showed that differentially expressed genes can be divided into six clusters.

FIGURE S2 The C1EIP located in cytoplasmic. **(A)** The EGFP fusion protein expression in DF-1 cells. Cells were transfected with pEGFP-N1 vector as a positive control, or mock-transfected with double distilled water (DDW) as a negative control. Scale bar: 200 μm. **(B)** The Western Blot analysis of EGFP fusion protein expression in DF-1 cells. M: Protein Marker; 1: Cells were transfected with pEGFP-N1 vector; 2: Cells were transfected with pEGFP-C1EIP vector; 3: mock-transfected with double distilled water (DDW). **(C)** IFA results show that the polyclonal antibody titer, Scale bar: 100 μm. **(D)** The Western Blot analysis of C1EIP polyclonal antibody. M: Protein Marker; 1: Cells were transfected with pcDNA3.1(+) vector; 2: Cells were transfected with pcDNA3.1-C1EIP vector; 3: mock-transfected with double distilled water (DDW). **(E)** The Western Blot analysis of negative polyclonal antibody. M: Protein Marker; 1: Cells were transfected with pcDNA3.1(+) vector; 2: Cells were transfected with pcDNA3.1-C1EIP vector; 3: mock-transfected with double distilled water (DDW).

FIGURE S3 Construction of interference vectors. **(A)** Schematic diagram of shRNAs targeting at C1EIP loci. **(B)** Photographs of DF1 cell line transfected by lentiviral shRNA (100×). Scale, 125 μm. **(C)** The expression of C1EIP gene in lentiviral-mediated RNA interference in DF1 cell line. Lentiviral interference vectors C1EIP-sh1∼C1EIP-sh3 and C1EIP-shCtrl infected DF1 cells, which were then treated with puromycin to screen positive cells. After 24 h, qRT-PCR was performed to detect C1EIP knockdown effect. The knockdown efficiency of C1EIP-sh1, C1EIP-sh2 and C1EIP-sh3 on C1EIP were 80.39, 87.80, and 38.36%, respectively (data was shown as mean ± SEM and student *t*-test were utilized for statistical analysis; ^∗^*p* < 0.05, ^∗∗^*p* < 0.01).

FIGURE S4 C1EIP enhances the PGC generation in vitro. **(A)** Antibody-specific detection of CVH by flow cytometry. **(B)** The morphological changes of ESCs in each group (400×). **(C)** qRT-PCR was used to quantify *Intergrin α6, Intergrin β1, Dazl*, and *Stra8* expression after C1EIP knockout or overexpression (data was shown as mean ± SEM and student *t*-test were utilized for statistical analysis; ^∗^*p* < 0.05, ^∗∗^*p* < 0.01).

FIGURE S5 Exogenous reporter vectors are integrated and expressed in chicken embryos. **(A)** Stereotactic fluorescence microscopy was used to observe the stable expression of exogenous EGFP-expressing reporter vector during chicken embryo development. Chicken embryos that had not been injected with an exogenous vector were used as controls, Scale bar: 5 mm. **(B)** Frozen sections of 4.5-day-old chicken embryos showing EGFP expression from an exogenous reporter vector, Scale bar: 130 μm.

TABLE S1 The components of the medium.

TABLE S2 The primes for qRT-PCR.

TABLE S3 The primers of C1EIP differential deletion clone fragments.

TABLE S4 The primers of transcriptional binding factors for PCR amplification.

TABLE S5 Target sites sequence of C1EIP gene.

## Abbreviations

5-AZA: 5-aza-2 ′ -deoxycytidine DNA methyltransferase inhibitors
C1EIP: chromosome 1 expression in PGCs
C2EIP: chromosome 2 expression in PGCs
CKIT: chicken KIT proto-oncogene
CVH: chicken vasa homologous
ENO1: enolase 1
ESCs: embryonic stem cells
FDR: false discovery rate
HH: hedgehog
KD: knockdown
Myc: MYC proto-oncogene
OE: overexpression
PAS: periodic acid Schiff
PGCs: primordial germ cells
qRT-PCR: quantitative real-time polymerase chain reaction
RA: retinoic acid
RPKM: reads per kb transcriptome per million reads
SSCs: spermatogonial stem cells
STAT3: signal transducer and activator of transcription 3
TSA, trichostatin: histone deacetylase inhibitor.
VPA, valproic acid: HDAC(Acetylation)inhibitor.
