# Supplementary figures and images for: C1EIP Functions as an Activator of ENO1 to Promote Chicken PGCs Formation via Inhibition of the Notch Signaling Pathway
Source: Front Genet. 2020 Jul 24;11:751. doi: 10.3389/fgene.2020.00751 (PMC7396672; doi:10.3389/fgene.2020.00751)

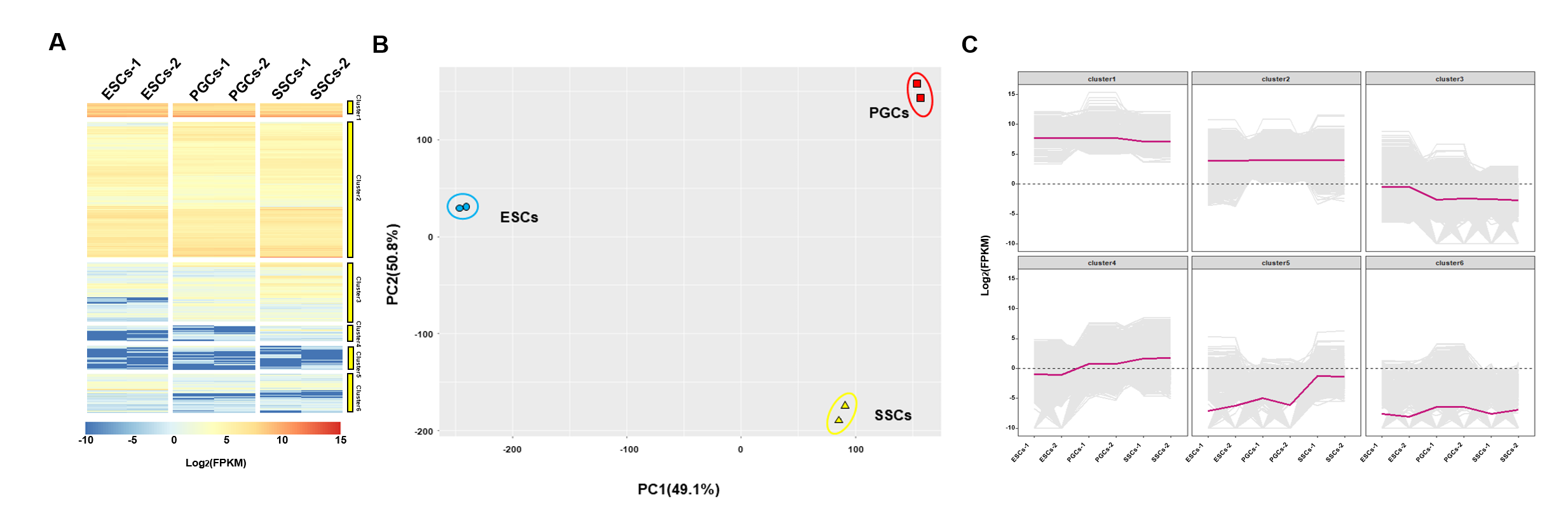

Supplement: FIGURE S1 — The C1EIP highly expressed in PGCs. (A) Transcriptome analysis of ESCs, PGCs, and SSCs. The differential expression of genes in these cells divided into six clusters. (B) The PCA analysis of C1EIP in ESC, PGC, and SSC. (C) The line chart showed that differentially expressed genes can be divided into six clusters. [file Image_1.png]

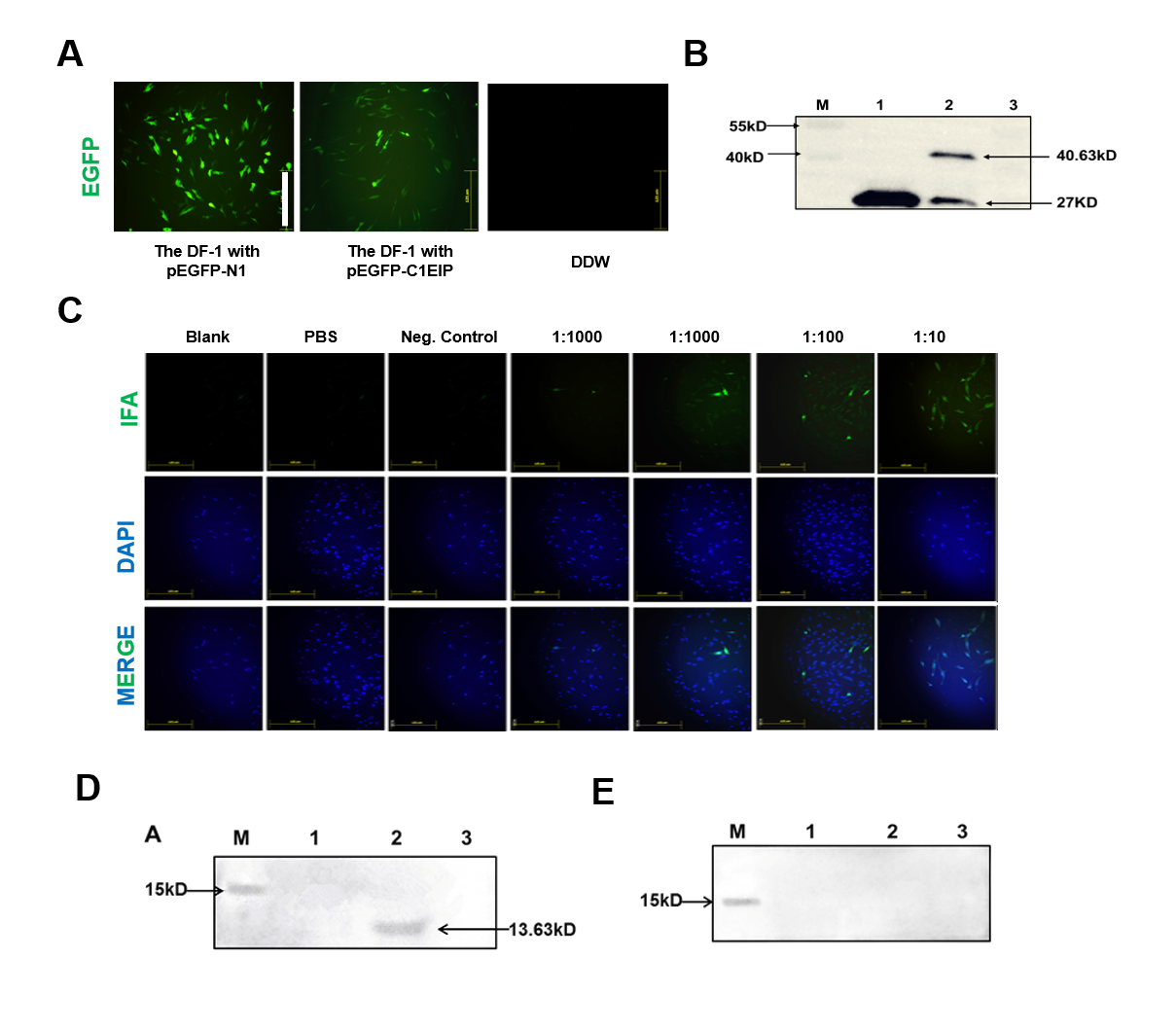

Supplement: FIGURE S2 — The C1EIP located in cytoplasmic. (A) The EGFP fusion protein expression in DF-1 cells. Cells were transfected with pEGFP-N1 vector as a positive control, or mock-transfected with double distilled water (DDW) as a negative control. Scale bar: 200 μm. (B) The Western Blot analysis of EGFP fusion protein expression in DF-1 cells. M: Protein Marker; 1: Cells were transfected with pEGFP-N1 vector; 2: Cells were transfected with pEGFP-C1EIP vector; 3: mock-transfected with double distilled water (DDW). (C) IFA results show that the polyclonal antibody titer, Scale bar: 100 μm. (D) The Western Blot analysis of C1EIP polyclonal antibody. M: Protein Marker; 1: Cells were transfected with pcDNA3.1(+) vector; 2: Cells were transfected with pcDNA3.1-C1EIP vector; 3: mock-transfected with double distilled water (DDW). (E) The Western Blot analysis of negative polyclonal antibody. M: Protein Marker; 1: Cells were transfected with pcDNA3.1(+) vector; 2: Cells were transfected with pcDNA3.1-C1EIP vector; 3: mock-transfected with double distilled water (DDW). [file Image_2.png]

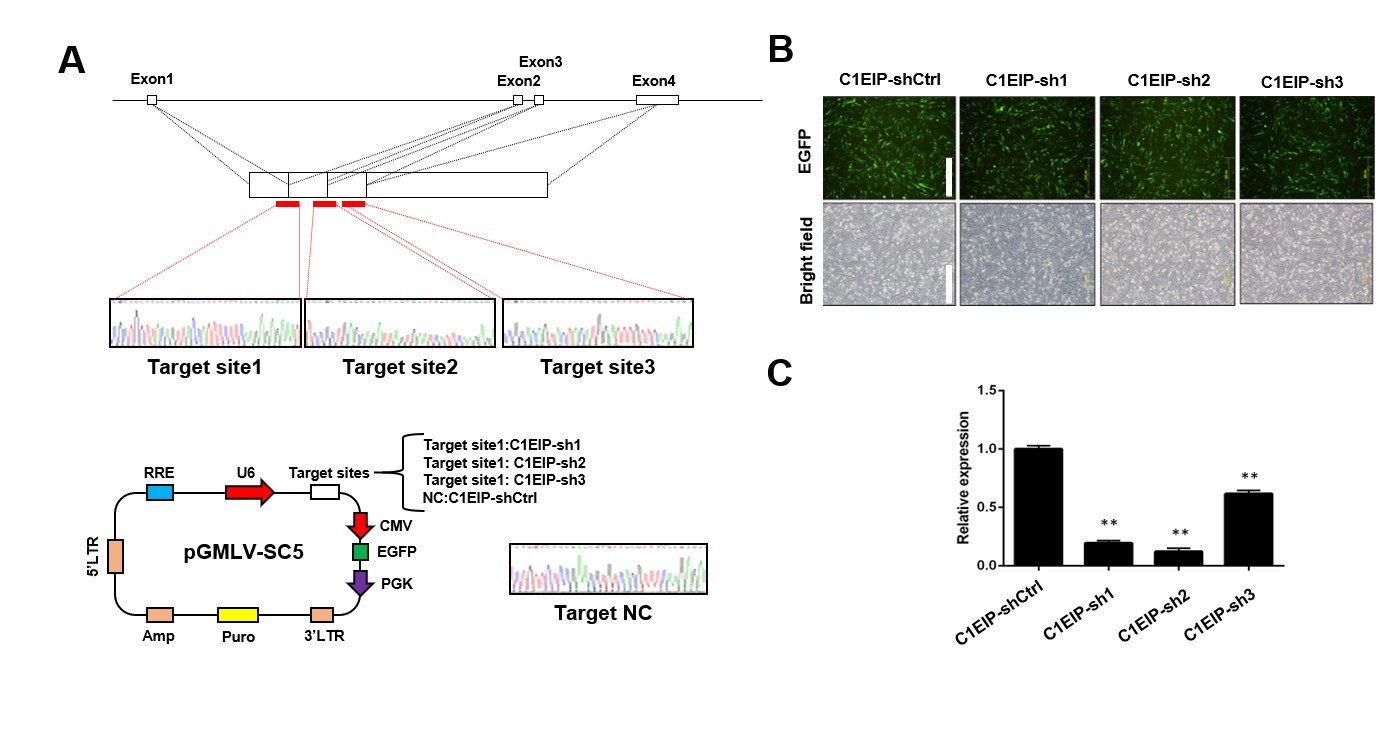

Supplement: FIGURE S3 — Construction of interference vectors. (A) Schematic diagram of shRNAs targeting at C1EIP loci. (B) Photographs of DF1 cell line transfected by lentiviral shRNA (100×). Scale, 125 μm. (C) The expression of C1EIP gene in lentiviral-mediated RNA interference in DF1 cell line. Lentiviral interference vectors C1EIP-sh1∼C1EIP-sh3 and C1EIP-shCtrl infected DF1 cells, which were then treated with puromycin to screen positive cells. After 24 h, qRT-PCR was performed to detect C1EIP knockdown effect. The knockdown efficiency of C1EIP-sh1, C1EIP-sh2 and C1EIP-sh3 on C1EIP were 80.39, 87.80, and 38.36%, respectively (data was shown as mean ± SEM and student t-test were utilized for statistical analysis; ∗p < 0.05, ∗∗p < 0.01). [file Image_3.png]

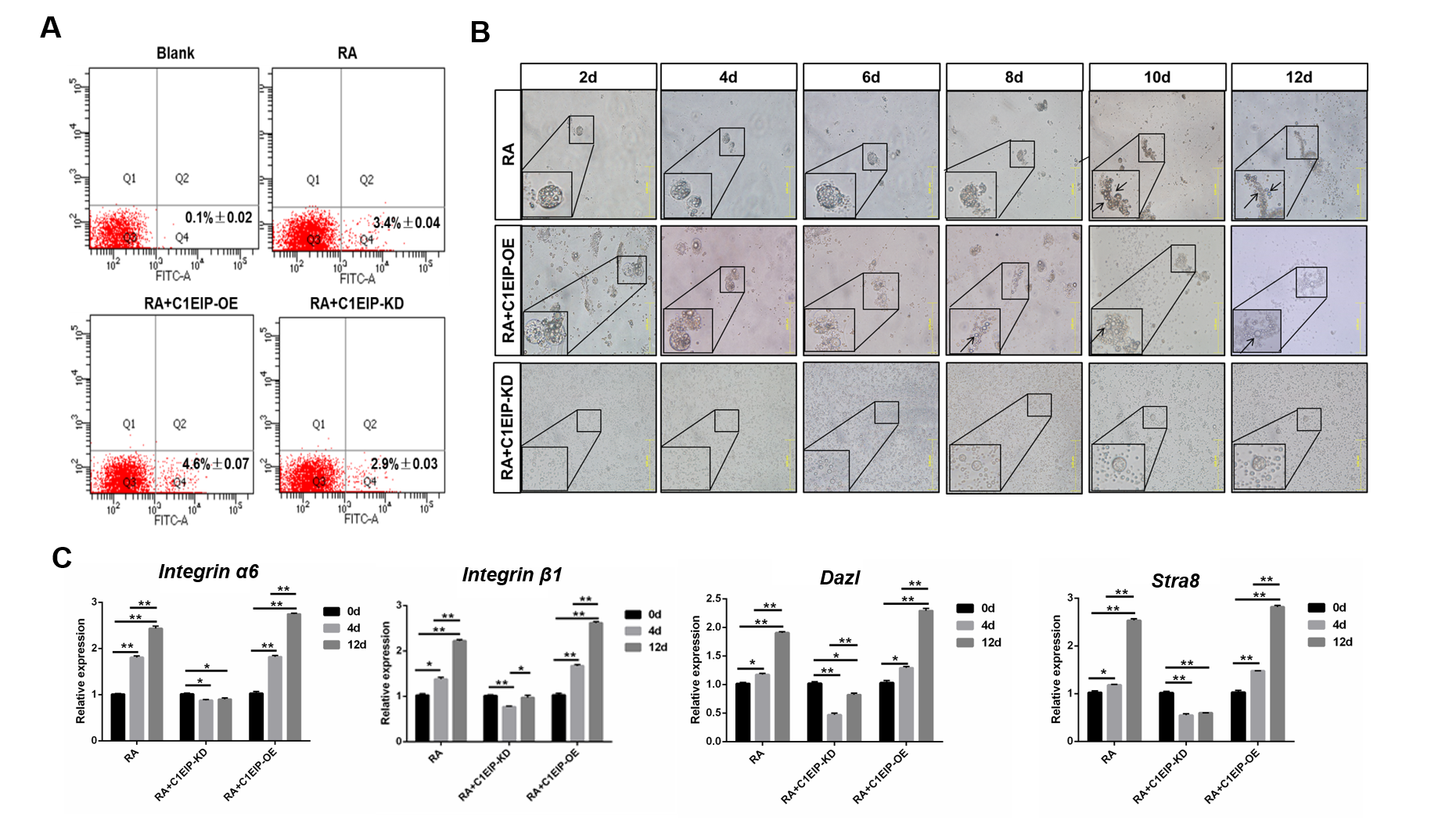

Supplement: FIGURE S4 — C1EIP enhances the PGC generation in vitro. (A) Antibody-specific detection of CVH by flow cytometry. (B) The morphological changes of ESCs in each group (400×). (C) qRT-PCR was used to quantify Intergrin α6, Intergrin β1, Dazl, and Stra8 expression after C1EIP knockout or overexpression (data was shown as mean ± SEM and student t-test were utilized for statistical analysis; ∗p < 0.05, ∗∗p < 0.01). [file Image_4.png]

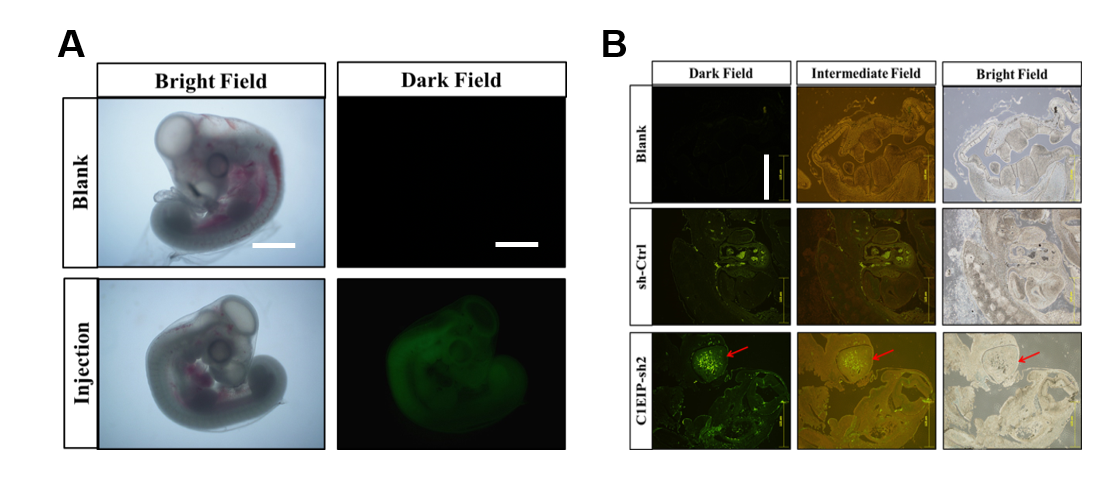

Supplement: FIGURE S5 — Exogenous reporter vectors are integrated and expressed in chicken embryos. (A) Stereotactic fluorescence microscopy was used to observe the stable expression of exogenous EGFP-expressing reporter vector during chicken embryo development. Chicken embryos that had not been injected with an exogenous vector were used as controls, Scale bar: 5 mm. (B) Frozen sections of 4.5-day-old chicken embryos showing EGFP expression from an exogenous reporter vector, Scale bar: 130 μm. [file Image_5.png]
