# Supplementary material for: C1EIP Functions as an Activator of ENO1 to Promote Chicken PGCs Formation via Inhibition of the Notch Signaling Pathway
Source: Front Genet. 2020 Jul 24;11:751. doi: 10.3389/fgene.2020.00751 (PMC7396672; doi:10.3389/fgene.2020.00751)
Supplement: TABLE S1 — The components of the medium. [file Table_1.docx]

**Supplementary Table1** The components of the medium

| Cell type | Medium Components |
| --- | --- |
| ESCs | DMEM (43.5ml) + FBS (15ml) + Gentamicin (100μl) + β-mercaptoethanol (0.2μl) + non-essential amino acids (200μl) + chicken serum (1ml) + SCF (100μl) + bFGF (100μl) + LIF (100μl) + Penicillin (500μl) |
| PGCs | DMEM (43.5ml) + FBS (15ml) + Gentamicin (100μl) + β-mercaptoethanol (0.2μl) + non-essential amino acids (200μl) + chicken serum (1ml) + SCF (100μl) + bFGF (100μl) + LIF (100μl) + Penicillin (500μl) + IL11(100μl) |
| SSCs | DMEM (43.5ml) + FBS (15ml) + Gentamicin (100μl) + β-mercaptoethanol (0.2μl) + non-essential amino acids (200μl) + chicken serum (1ml) + SCF (100μl) + bFGF (100μl) + LIF (100μl) + Penicillin (500μl) + GDNF (100ul) |
