# Supplementary material for: C1EIP Functions as an Activator of ENO1 to Promote Chicken PGCs Formation via Inhibition of the Notch Signaling Pathway
Source: Front Genet. 2020 Jul 24;11:751. doi: 10.3389/fgene.2020.00751 (PMC7396672; doi:10.3389/fgene.2020.00751)
Supplement: TABLE S2 — The primes for qRT-PCR. [file Table_2.docx]

**Supplementary Table2** The primes for qRT-PCR

| Gene | Primer (5’-3’) | | length（bp） |
| --- | --- | --- | --- |
| *sox2* | FOR | GAAGATGCACAACTCGGAGATCAG | 100 |
|  | REV | GAGCCGTTTGGCTTCGTCA |  |
| *cvh* | FOR | AGGAGGACTGGGACACG | 164 |
|  | REV | GCCTCTTGATGCTACCG |  |
| *c-kit* | FOR | GCGAACTTCACCTTACCCGATTA | 150 |
|  | REV | TGTCATTGCCGAGCATATCCA |  |
| *stra8* | FOR | TGATGGCGATGTGAGGGA | 115 |
|  | REV | AAGGAAACCAGCAGCAAC |  |
| *Dazl* | FOR | TGTCTTGAAGGCCTCGTTTG | 138 |
|  | REV | CATATCCTTGGCAGGTTGTTGA |  |
| *integrin α6* | FOR | AGATTTACCTATTTACCGATTG | 117 |
|  | REV | CTGTGGATTTCTTGCGTC |  |
| *integrin β1* | FOR | TGTTTGTGGGGACCAGATTG | 120 |
|  | REV | CCAGGTGACATTTCCCATCA |  |
| *C1EIP* | FOR | ATCTGCTCCAGGTTTGGCTA | 104 |
|  | REV | TGGCAGGTTTAGTTTCAGTCA |  |
| *ENO1* | FOR | CGGTGCCTCAACTGGAAT | 193 |
|  | REV | CTCCGTTCCATCCATTTCC |  |
| *Myc* | FOR | GCCAGCGAAGGAATGAGC | 176 |
|  | REV | CCGCCTCAACTGCTCTTTCT |  |
| *Notch1* | FOR | AAGGACCCGTATCAGC | 293 |
|  | REV | TCAGGAGGTGGGAAGT |  |
| *β-actin* | FOR | CAGCCATCTTTCTTGGGTAT | 164 |
|  | REV | CTGTGATCTCCTTCTGCATCC |  |
