# Supplementary material for: C1EIP Functions as an Activator of ENO1 to Promote Chicken PGCs Formation via Inhibition of the Notch Signaling Pathway
Source: Front Genet. 2020 Jul 24;11:751. doi: 10.3389/fgene.2020.00751 (PMC7396672; doi:10.3389/fgene.2020.00751)
Supplement: TABLE S3 — The primers of C1EIP differential deletion clone fragments. [file Table_3.docx]

**Supplementary Table3** The primers of C1EIP differential deletion clone fragments

| Primer | Primer sequence（5’-3’） |
| --- | --- |
| pGL3-P1  (-1963~-90) | F：GGGGTACCTTCCTTCCACCTTTAATAATAG  R：CCGCTCGAGCCAGATACCTGTGAACCG |
| pGL3-P2  (-1667~-90) | F： GGGGTACCATAGCTGTGAATTGGGTTACT R： CCGCTCGAGCCAGATACCTGTGAACCG |
| pGL3-P3  (-1376~-90) | F： GGGGTACCCTAAAGATGTGTCACTAAATTCC  R： CCGCTCGAGCCAGATACCTGTGAACCG |
| pGL3-P4  (-1025~-90) | F： GGGGTACCGGCATTACTTTCAGGGTG R： CCGCTCGAGCCAGATACCTGTGAACCG |
| pGL3-P5  (-410~-90) | F：GGGGTACCTTCAAACACCATTTCCCA  R：CCGCTCGAGCCAGATACCTGTGAACCG |
| pGL3-P4.1  (-912~-90) | F： GGGGTACCAAATACTGCTGTCCATCACC  R： CCGCTCGAGCCAGATACCTGTGAACCG |
| pGL3-P4.2  (-799 ~ -90) | F： GGGGTACCTCCTAATCATCTATCGCATCC R： CCGCTCGAGCCAGATACCTGTGAACCG |
| pGL3-P4.3  (-671~-90) | F：GGGGTACCTATACGAAAAGGCAAAATTTA  R：CCGCTCGAGCCAGATACCTGTGAACCG |
| pGL3-P4.4  (-584~-90) | F：GGGGTACCTAGAAGAGGACAACAGGAGAC R：CCGCTCGAGCCAGATACCTGTGAACCG |
| pGL3-P4.5  (-472~-90) | F：GGGGTACCATGCAAATATTATATAGCTTAAATA  R：CCGCTCGAGCCAGATACCTGTGAACCG |
