# Supplementary material for: C1EIP Functions as an Activator of ENO1 to Promote Chicken PGCs Formation via Inhibition of the Notch Signaling Pathway
Source: Front Genet. 2020 Jul 24;11:751. doi: 10.3389/fgene.2020.00751 (PMC7396672; doi:10.3389/fgene.2020.00751)
Supplement: TABLE S4 — The primers of transcriptional binding factors for PCR amplification. [file Table_4.docx]

**Supplementary Table4** The primers of transcriptional binding factors for PCR amplification

| Primer | Primer Squences（5’-3’） |
| --- | --- |
| MEIS1& MAFG（mut） | F： CTATGTACATATCCATTTTCTTCCAAGCAAACTTTTGC  R： CTTGGAAGAAAATGGATATGTACATAGGTCACCCTGAAAG |
| STAT3（mut） | F： CCAAATGACATTTTTACTTTTGCTCAGTACACAGTC R： GAGCAAAAGTAAAAATGTCATTTGGATATGTACATAG |
| HLTF（mut） | F：CATTTTTCTTCCAAGCTCAGTACACAGTCTGGAATGAATG R： GACTGTGTACTGAGCTTGGAAGAAAAATGTCATTTGGAT |
| Hand1::Tcf3（mut） | F： CTTTTGCTCAGTACACGAATGGCTCTACTGCAAGTGTAAG R： GTAGAGCCATTCGTGTACTGAGCAAAAGTTTGCTTGGAAG |
