# Supplementary material for: C1EIP Functions as an Activator of ENO1 to Promote Chicken PGCs Formation via Inhibition of the Notch Signaling Pathway
Source: Front Genet. 2020 Jul 24;11:751. doi: 10.3389/fgene.2020.00751 (PMC7396672; doi:10.3389/fgene.2020.00751)
Supplement: TABLE S5 — Target sites sequence of C1EIP gene. [file Table_5.docx]

**Supplementary Table5** Target sites sequence of C1EIP gene

| Site. | Target Sequence |
| --- | --- |
| Sh-1 | GCTGCTTACAGTTACCATTTC |
| Sh-2 | GCTGAGCAGATTAGTGAATCA |
| Sh-3 | GCACAGTACTACATGGATTTG |
| NC | TTCTCCGAACGTGTCACGT |
